# Microfluidic devices as model platforms of CNS injury-ischemia to study axonal regeneration by regulating mitochondrial transport and bioenergetic metabolism

**DOI:** 10.1186/s13619-022-00138-3

**Published:** 2022-10-03

**Authors:** Ning Huang, Zu-Hang Sheng

**Affiliations:** 1 Synaptic Function Section, The Porter Neuroscience Research Center, National Institute of Neurological Disorders and Stroke, National Institutes of Health, Room 2B-215, 35 Convent Drive, Bethesda, MD 20892-3706 USA; 2 Department of Physiology and Pathophysiology, School of Basic Medical Sciences, Xi’an Jiaotong University Health Science Center, Xi’an, 710061 Shaanxi China; 3 Institute of Neuroscience, Translational Medicine Institute, Xi’an Jiaotong University Health Science Center, Xi’an, 710061 Shaanxi China

**Keywords:** Microfluidic device, Axon injury, Ischemia, Axon regeneration, Mitochondrial transport, Axonal bioenergetics, Axonal protein synthesis

## Abstract

Central nervous system (CNS) neurons typically fail to regenerate their axons after injury leading to neurological impairment. Axonal regeneration is a highly energy-demanding cellular program that requires local mitochondria to supply most energy within injured axons. Recent emerging lines of evidence have started to reveal that injury-triggered acute mitochondrial damage and local energy crisis contribute to the intrinsic energetic restriction that accounts for axon regeneration failure in the CNS. Characterizing and reprogramming bioenergetic signaling and mitochondrial maintenance after axon injury-ischemia is fundamental for developing therapeutic strategies that can restore local energy metabolism and thus facilitate axon regeneration. Therefore, establishing reliable and reproducible neuronal model platforms is critical for assessing axonal energetic metabolism and regeneration capacity after injury-ischemia. In this focused methodology article, we discuss recent advances in applying cutting-edge microfluidic chamber devices in combination with state-of-the-art live-neuron imaging tools to monitor axonal regeneration, mitochondrial transport, bioenergetic metabolism, and local protein synthesis in response to injury-ischemic stress in mature CNS neurons.

## Background

Although developing CNS neurons show robust axon growth, mature neurons typically fail to regrow after injury, leading to permanent neurological impairments. Axon regeneration is a complicated cellular program that includes axonal protein synthesis, cytoskeleton reorganization, organelle transport, and synapse re-formation (Bradke et al. 2012; He and Jin 2016; Lu et al. 2014). All these processes require high levels of energy consumption. Mitochondria are the main cellular power plants in neurons, where mitochondria generate energy in the form of ATP to power neuron growth, survival, and repair (Sheng 2017). Due to the highly polarized structures of neurons with a long axon and extensive branches and terminals, and limited ATP diffusion within axons (Sun et al. 2013), neurons face exceptional challenges in trafficking and positioning mitochondria to distal axons in order to maintain local energy supply (see review by Li and Sheng 2022). This is particularly problematic following CNS injuries, such as spinal cord injury (SCI) and ischemia, that acutely damage local mitochondria leading to an energy crisis in injured axons. Recent studies provide multiple lines of evidence suggesting that injury-induced mitochondrial damage is an intrinsic barrier limiting local energy supply that accounts for regeneration failure in the CNS. Thus, enhancing mitochondrial transport not only removes those damaged mitochondria within injured axons but also replenishes with healthy ones to recover energy supply to power regenerative events (see review by Cheng et al. 2022). Characterizing and reprogramming axonal mitochondrial trafficking and repairing the local energy crisis represent an emerging research frontier in the CNS axon regeneration.

It is particularly urgent to elucidate cellular mechanisms and intrinsic signaling pathways that regulate mitochondrial transport and/or boost energy metabolism to meet enhanced energy consumption in response to injury and ischemia. Pursuing these investigations will conceptually advance knowledge of axonal energy deficits in CNS injuries and neurological disorders and help determine whether energy repairing programs facilitate CNS regeneration. Thus, establishing an in vitro injury-ischemia model platform that ensures reliable, reproducible, and robust approaches is critical for monitoring axonal mitochondrial transport, assessing axonal bioenergetic status, and analyzing axonal regenerative capacity. The recent development of microfluidic chamber devices provides a valuable platform to physically and fluidically separate axons from somas and dendrites of cultured CNS neurons, thus allowing live measurement of mitochondrial transport, dynamic energy metabolism, and local signaling processes and protein synthesis within injured axonal compartments (Han et al. 2020; Huang et al. 2021; Neto et al. 2016; Zhou et al. 2016). In this focused methodology article, we discuss this state-of-art microfluidic chamber-based platform that provides a model system for acute CNS axonal injury in vitro. We also present imaging approaches to (1) detect dynamic changes in cellular ATP levels within axon bundles and regrowing tips, (2) monitor axonal mitochondrial motility and energy metabolism, and (3) visualize local mitochondrial protein synthesis *in situ* in response to injury and ischemic stress in mature CNS neurons.

## Results

### Using microfluidic devices to model CNS axon regeneration and degeneration after injury and ischemia

**Fig. 1:**
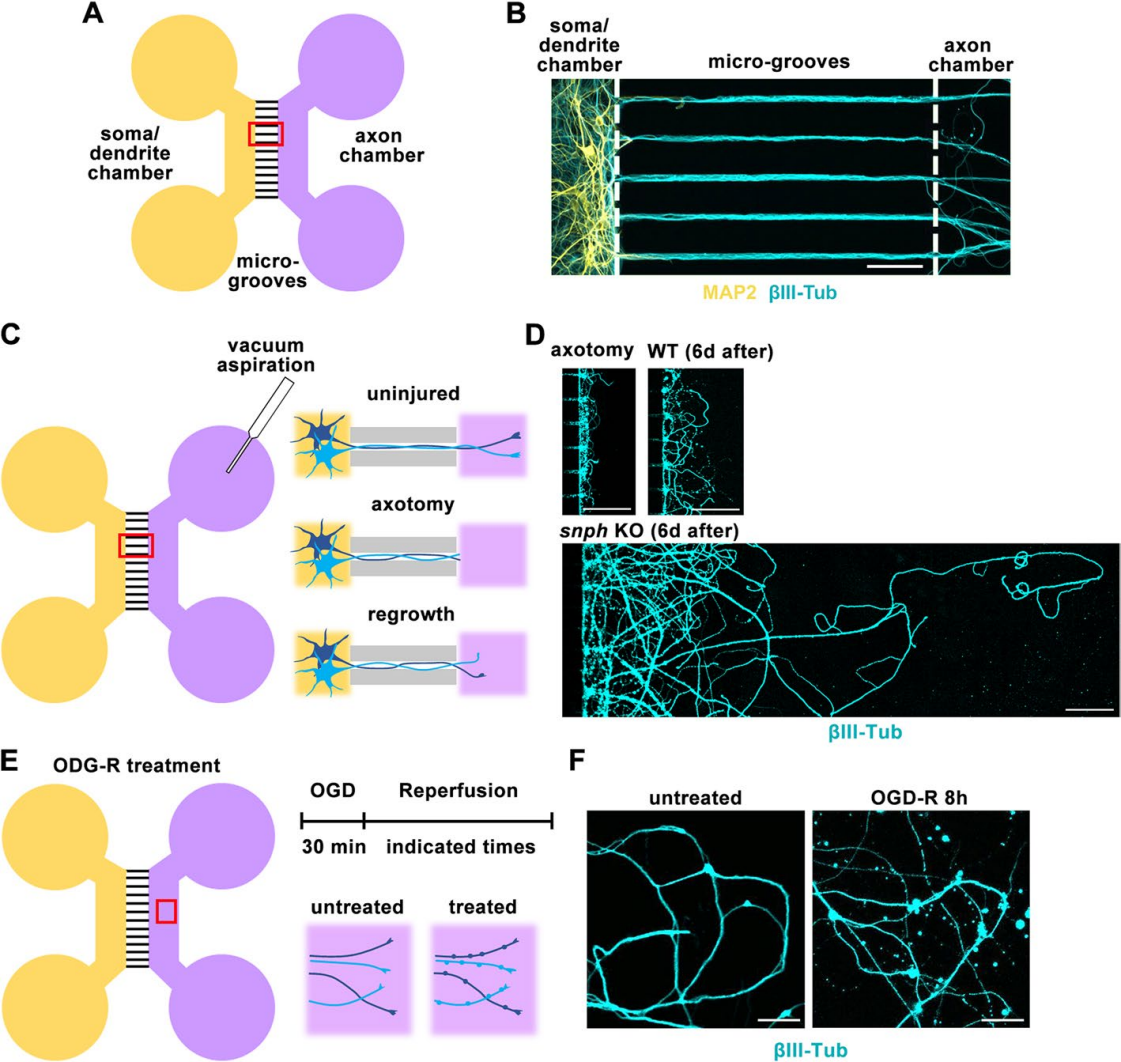
Using microfluidic chamber devices to model CNS axon regeneration and degeneration after injury and ischemia. **A** Schematic of a microfluidic chamber device that physically and fluidically separates axons from soma-dendritic area of neurons. Cortical neurons were plated in the soma-dendritic chamber (yellow) at DIV0, and only axons grow into the axon terminal chamber (purple) through the 450-μm microgrooves (red box). **B** Co-immunostaining validates axon compartments (labeled by βIII-tubulin) and soma-dendritic compartments (labeled by MAP2). **C**, **D** Schematic showing axotomy via vacuum aspiration within axon chambers (**C**) and representative images of axon chambers showing relative axonal regrowth capacity 6 days post injury (**D**). WT or *snph* KO cortical neurons were axotomized at DIV10 and labeled with βIII-tubulin for imaging 6 days post axotomy. **E**, **F** Schematic showing OGD-R treatment within axonal chamber (**E**) and representative images of axon chambers showing axonal integrity before (left) or after (right) OGD-R treatment. Cortical neurons at DIV14 were treated with OGD for 30 minutes and reperfusion for 8 hours, followed by immunostaining with βIII-tubulin. Note that the ischemic stress induces bead-like or fragmented axons. Scale bars, 50 μm (**B**), 100 μm (**D**), 20 μm (**F**). Imaging (**B**) is adapted from Farfel-Becker et al. 2019 with permission

Microfluidic chamber devices provide a powerful tool to physically and fluidically separate axons from neuronal somas and dendrites (Zhou et al. 2012). Upon plating in the soma chamber of the microfluidic devices, neuronal cell bodies and dendrites are restricted to the soma chamber while axons grow into the axon terminal chamber through long microgrooves (450 μm in length) (Zhou et al. 2016) (Fig. 1A, B). To establish an in vitro model for CNS axon injury and regeneration, we applied vacuum aspiration to remove the axons of mouse cortical neurons at 14 days in vitro (DIV14), as previously reported (Fig. 1C) (Taylor et al. 2005). Immunostaining validates that this vacuum axotomy method effectively cuts off all axons at the entrance of the axon terminal chamber and that lesioned axons from mature CNS neurons display limited regrowth capacity 6 days after injury (Fig. 1D). However, enhancing axonal mitochondrial transport by deleting the mitochondrial anchor syntaphilin (SNPH) robustly facilitated axon regeneration (Fig. 1D) (Huang et al. 2021). These regenerative data from the microfluidic platform are consistent with our in vivo observations in *snph* knockout (KO) mice, which display enhanced sciatic nerve regeneration after crush injury (Zhou et al. 2016) and enhanced corticospinal tract (CST) axonal regeneration and motor function recovery after SCI (Han et al. 2020). Thus, both in vitro and in vivo injury model systems support the notion that facilitating axonal mitochondrial transport helps recover local energy supply, thus powering CNS regeneration (see review by Cheng et al. 2022).

To model ischemic stress and subsequent axonal degeneration in CNS neurons, we modified an ischemic-reperfusion condition (Zheng et al. 2019) by treating mature (DIV14) cortical neurons in microfluidic devices with oxygen-glucose-deprivation medium (OGD: 1% O_2_, 5% CO_2_ and 94% N_2_, and glucose-free) for 30 min, followed by reperfusion (R) for 8 hours with oxygen-glucose-containing medium (Fig. 1E). Mature neurons subjected to the OGD-R treatment displayed typical degenerative phenotypes, including bead-like or fragmented axons (Fig. 1F) (Huang et al. 2021), consistent with the ischemia-induced axon degeneration phenotype reported in cultured neurons and mouse brain white matter tracts after ischemia (Cui et al. 2020; Liu et al. 2019). These experimental platforms using microfluidic chamber devices provide reliable model systems for studying CNS axon regeneration after injury and ischemia.

### Measurement of axonal ATP levels before and after injury and ischemia

**Fig. 2:**
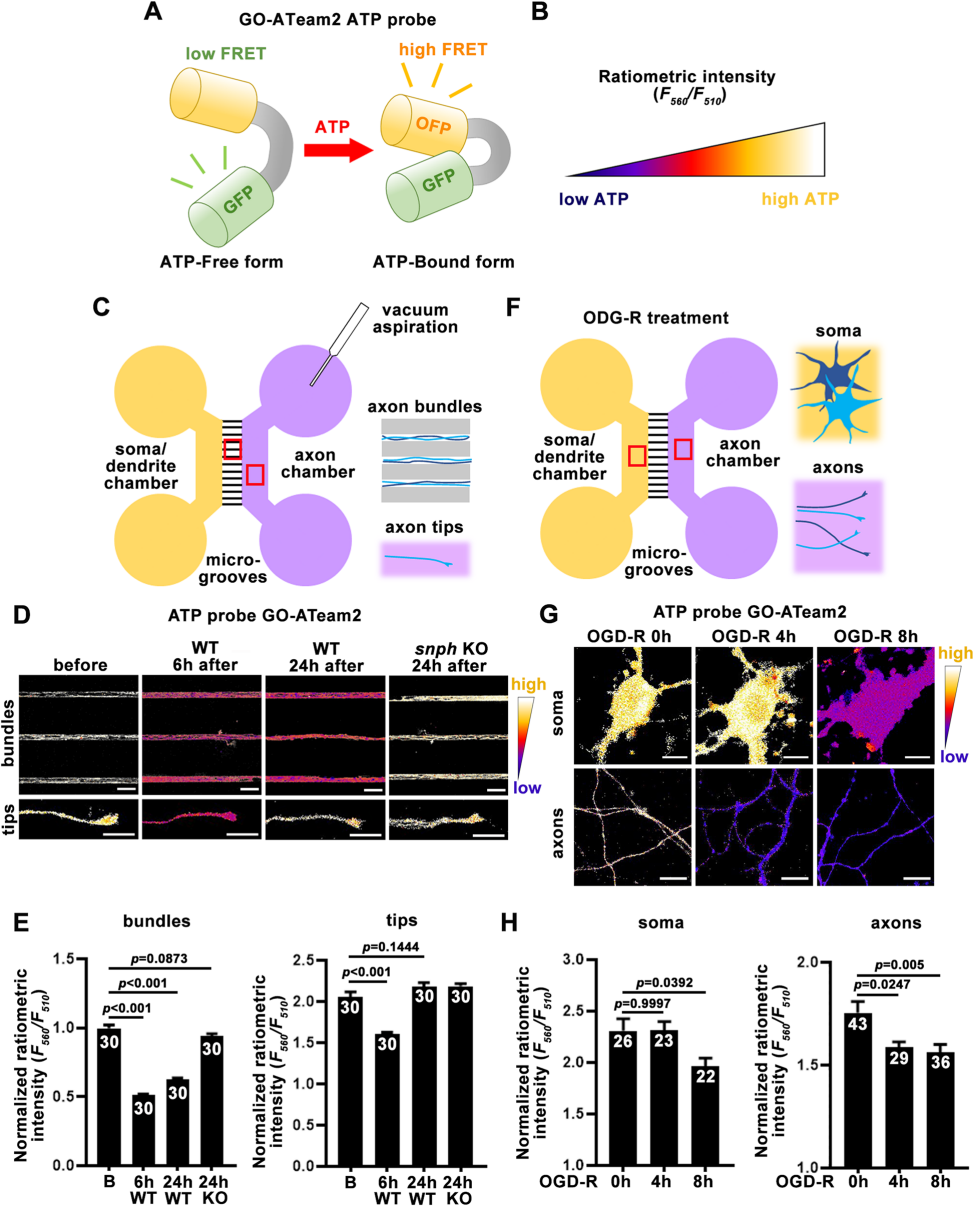
Measurement of axonal ATP levels before and after injury and ischemia. **A**, **B** Schematic (**A**) and color-coded heatmap index (**B**) of the GO-ATeam2 ATP probe. GO-ATeam2 is a FRET-based and red-shifted ATP sensor (Nakano et al. 2011) that emits at 560 nm (OFP) when it is bound to ATP, and 510 nm (GFP) when it is free from ATP, and thus higher 560 nm/510 nm intensity ratios indicate relatively higher intracellular ATP levels. **C**-**E** Schematic (**C**), representative images (**D**), and quantifications (**E**) showing the dynamic changes in axonal ATP levels within axon bundles and growing tips after vacuum aspiration-based axotomy. WT or *snph* KO cortical neurons in microfluidic chambers were infected with lentiviruses encoding GO-ATeam2 and imaged at DIV14 before or at different time points post injury. Scale bars, 50 μm (bundles) and 10 μm (tips). **F**-**H** Schematic (**F**), representative images (**G**), and quantifications (**H**) showing the dynamic changes of cytosolic ATP levels within cell bodies and axons after the OGD-R treatment of cortical neurons. Cortical neurons were infected with lentiviruses encoding GO-ATeam2 and imaged at DIV14 before or at different time points after OGD-R. Scale bars, 10 μm. Data were quantified from *n* = 30 (**E**) or *n* = 22–43 images (**H**) from more than 3 chambers per condition in three independent experiments. Data were displayed as the mean ± SEM by one-way ANOVA with Tukey’s multiple comparisons test

Brain injury and ischemia damage local mitochondria leading to an energy crisis and the release of toxic and apoptotic factors, thus triggering neuronal death (see reviews by Sheng 2017; Vosler et al. 2009; Cheng et al. 2022). Recent studies have started to reveal that (1) injury-induced energetic restriction accounts for regeneration failure in the CNS and (2) recruiting healthy mitochondria into injured axons repairs bioenergetic deficits. To spatial-temporally monitor axonal energy dynamics within injured axons and regrowing tips, we measured intracellular ATP levels using an ATP sensor named GO-ATeam2. This red-shifted ATP sensor features a Förster Resonance Energy Transfer (FRET) pair between green fluorescent protein (GFP) and orange fluorescent protein (OFP), separated by a mutant ɛ subunit of F_0_F_1_-ATP synthase with higher ATP binding affinity (Nakano et al. 2011). In the ATP-free form, the extended, flexible ɛ subunit separates the two fluorescent proteins, resulting in low FRET efficiency and emission at 510 nm. In the ATP-bound form, the ɛ subunit retracts to draw the two fluorescent proteins close to each other to increase FRET efficiency, resulting in emission at 560 nm (Fig. 2A). Higher 560 nm/510 nm ratios indicate relatively higher ATP levels and are visually depicted using color-coded heatmaps (Fig. 2B).

We characterized the injury-induced energy crisis in mature cortical neurons by monitoring cytosolic ATP levels along axon bundles within microgrooves and at axonal regrowing tips in the axon chambers after vacuum aspiration-based axotomy (Fig. 2C). Relative ATP levels within axon bundles (*p* < 0.001) and tips (*p* < 0.001) significantly declined 6 hours after axotomy (Fig. 2D, E). However, at 24 hours post-axotomy, ATP levels in axon tips (*p* = 0.1444), but not axon bundles (*p* < 0.001), were largely recovered when compared to those before axotomy, reflecting an intrinsic capacity of CNS neurons to repair energy deficits. Consistently, enhancing axonal mitochondrial transport by deleting the mitochondrial anchor SNPH robustly recovered local energy levels within axon bundles (*p* = 0.0873, Fig. 2D, E) (Huang et al. 2021). We also examined the change in intracellular ATP levels in cortical neurons after OGD-R treatment (Fig. 2F). OGD-R treatment induced ATP depletion in both somas and axons. Interestingly, axonal ATP levels began to decrease at 4 hours after OGD-R (*p* = 0.0247), whereas ATP levels in the soma started to decline after 8 hours of OGD-R treatment (*p* = 0.0392, Fig. 2G, H), indicating that axonal energy levels are more vulnerable to ischemic stress.

### Live imaging of mitochondrial transport after axon injury and ischemia

In contrast to chronic and progressive neurodegeneration, brain injury and ischemia trigger acute mitochondrial damage leading to a local energy crisis. Thus, CNS neurons face exceptional challenges in trafficking healthy mitochondria from the soma to injured axons to replace damaged mitochondria and thus recover local energy supply. Mitochondrial transport and energy maintenance within injured axons are thus central problems in regeneration failure (Sheng 2017). In mature neurons and adult mouse brains, axonal mitochondrial anchor SNPH is highly expressed (Zhou et al. 2016), which anchors the majority of axonal mitochondria in a stationary status. Thus, SNPH becomes an attractive target for remobilizing and replacing injury-damaged mitochondria with healthy ones and thus recovering axonal bioenergetic status.

**Fig. 3:**
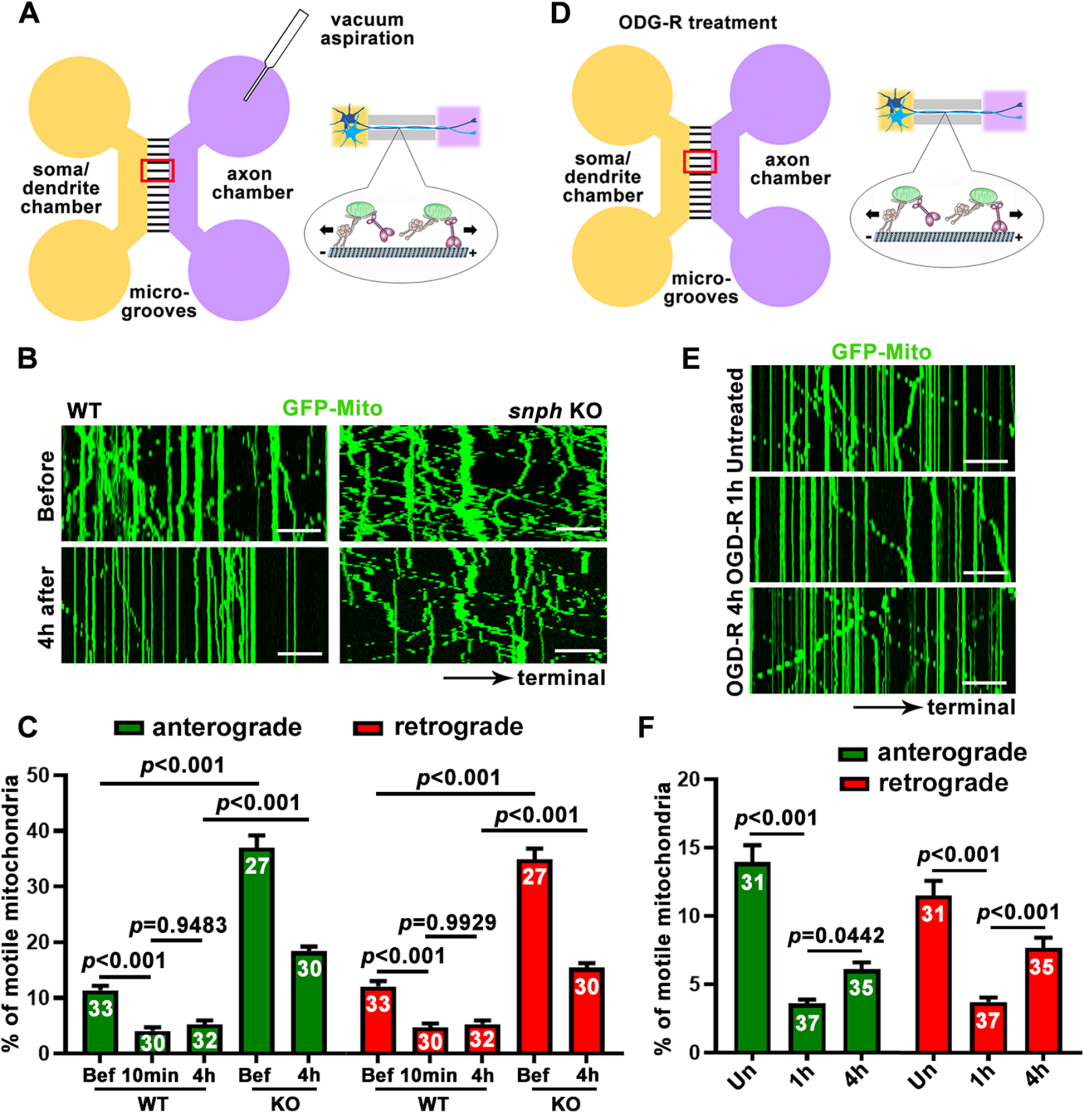
Live imaging of mitochondrial transport after axon injury and ischemia. Schematics (**A**, **D**) and representative kymographs (**B**, **E**) and quantifications (**C**, **F**) showing the bi-directional axonal mitochondrial transport after axotomy injury (**A-C**) or upon ischemia-reperfusion (**D**-**F**). Cortical neurons were infected with lentivirus encoding GFP-Mito, followed by live imaging at DIV14 before or post axotomy (**B**, **C**) or OGD-R treatment (**E**, **F**). Images were captured for 30 frames with 5-s intervals. In kymographs, vertical lines represent stationary mitochondria; oblique lines or curves to the right indicate anterograde transport toward distal terminals, whereas those to the left denote retrograde transport toward the cell body. Data were quantified from *n* = 27–37 microgrooves from more than 3 chambers per condition in three independent experiments. Data were expressed as mean ± SEM and analyzed by one-way ANOVA with Tukey’s multiple comparisons test. Scale bars, 10 μm

To test this hypothesis, we investigated (1) whether mitochondrial trafficking in mature neurons is largely abolished in response to local injury stress, thus blocking recovery of an energy crisis, and (2) whether reprogramming mitochondrial transport by deleting the *snph* gene can help replace damaged mitochondria and thus rescue local energy supply to support axon regrowth after injury. Using mature cortical neurons (DIV14) in microfluidic devices, we expressed GFP-Mito to label mitochondria and conducted live imaging to monitor axonal mitochondrial transport at different time points post axotomy (Fig. 3A). The relative motility (% motile of total mitochondria) before and 10 min or 4 hours post axotomy in mature cortical neurons were recorded along axon bundles within microgrooves, followed by kymograph analysis (Kang et al. 2008) (Fig. 3B, C). Bi-directional motility of axonal mitochondria significantly decreased at 10 min after injury (anterograde: from 11.36 ± 0.81% to 4.12 ± 0.61%, *p* < 0.001; retrograde: from 12.06 ± 0.97% to 4.70 ± 0.69%, *p* < 0.001), and such reduction was hardly recovered at 4 hours post axotomy (anterograde: 5.28 ± 0.68%, *p =* 0.95; retrograde: 5.31 ± 0.68%, *p =* 0.99) when compared to the motility at 10 min after injury. These data support the notion that acute brain injury induces an energy crisis in the vicinity of injured axons, where damaged mitochondria cannot be effectively removed and replaced by healthy ones (Zhou et al. 2016). We further demonstrated that deleting the *snph* gene remobilizes mitochondria in both directions before and after injury (Fig. 3B, C), and restores local energy supply (Fig. 2D, E), thus facilitating regenerative capacity after injury (Fig. 1D).

We also subjected mature cortical neurons to OGD treatment for 30 min, followed by reperfusion (R) with normal culture media for the indicated times (Fig. 3D). Compared with the untreated neurons (anterograde: 13.95 ± 1.25%; retrograde: 11.47 ± 1.08%), 1-hour OGD-R treatment remarkably impaired mitochondrial transport in both directions (anterograde: 3.62 ± 0.28%, *p* < 0.001; retrograde: 3.72 ± 0.31%, *p* < 0.001). In contrast to axotomy injury, axonal mitochondrial motility was partially recovered 4 hours post OGD-R treatment; retrograde transport showed more robust recovery than anterograde transport (anterograde: 6.09 ± 0.52%, *p* = 0.0442; retrograde: 7.66 ± 0.74%, *p* < 0.001, Fig. 3E, F) when compared with 1-hour post OGD-R treatment, which is consistent with a previous report (Zheng et al. 2019). This recovery pattern may reflect an intrinsic mechanism for ischemia-induced bioenergetic repair by favorably removing damaged mitochondria from distal axons for mitophagy in the soma, where mature lysosomes are relatively enriched. Although both axotomy injury and ischemic stress were reported to remobilize damaged stationary mitochondria within axons via AKT-PAK5-SNPH axis (Huang et al. 2021), transiently activation of this signaling axis by the two acute insults may have differential regulatory balance in controlling mitochondrial motors vs anchors.

### *In situ* visualization of axonal protein synthesis upon ischemic stress

Due to the highly polarized structure of neurons, injured axons need a specialized local mechanism to respond to the energy stress. Thus, local protein synthesis is one of the ideal solutions to effectively activate bioenergetic pathways within distal regenerative axons in response to acute axon injury. Recent studies revealed that mitochondria-linked signaling proteins, including LaminB2, VDAC2, PINK1, and PAK5, can be locally synthesized along axons (Cioni et al. 2019; Harbauer et al. 2022; Huang et al. 2021). Compared to the traditional pulse-chase labeling approaches in examining newly synthesized proteins, recent advances in metabolic labeling and proximity ligation assay (PLA) provide powerful imaging tools to visualize newly synthesized proteins *in situ* (tom Dieck et al. 2015). These cutting-edge tools have been elegantly applied to assess local protein synthesis in distal axonal compartments after axotomy in both the CNS and the peripheral nervous system (PNS) (Huang et al. 2021; Terenzio et al. 2018).

**Fig. 4:**
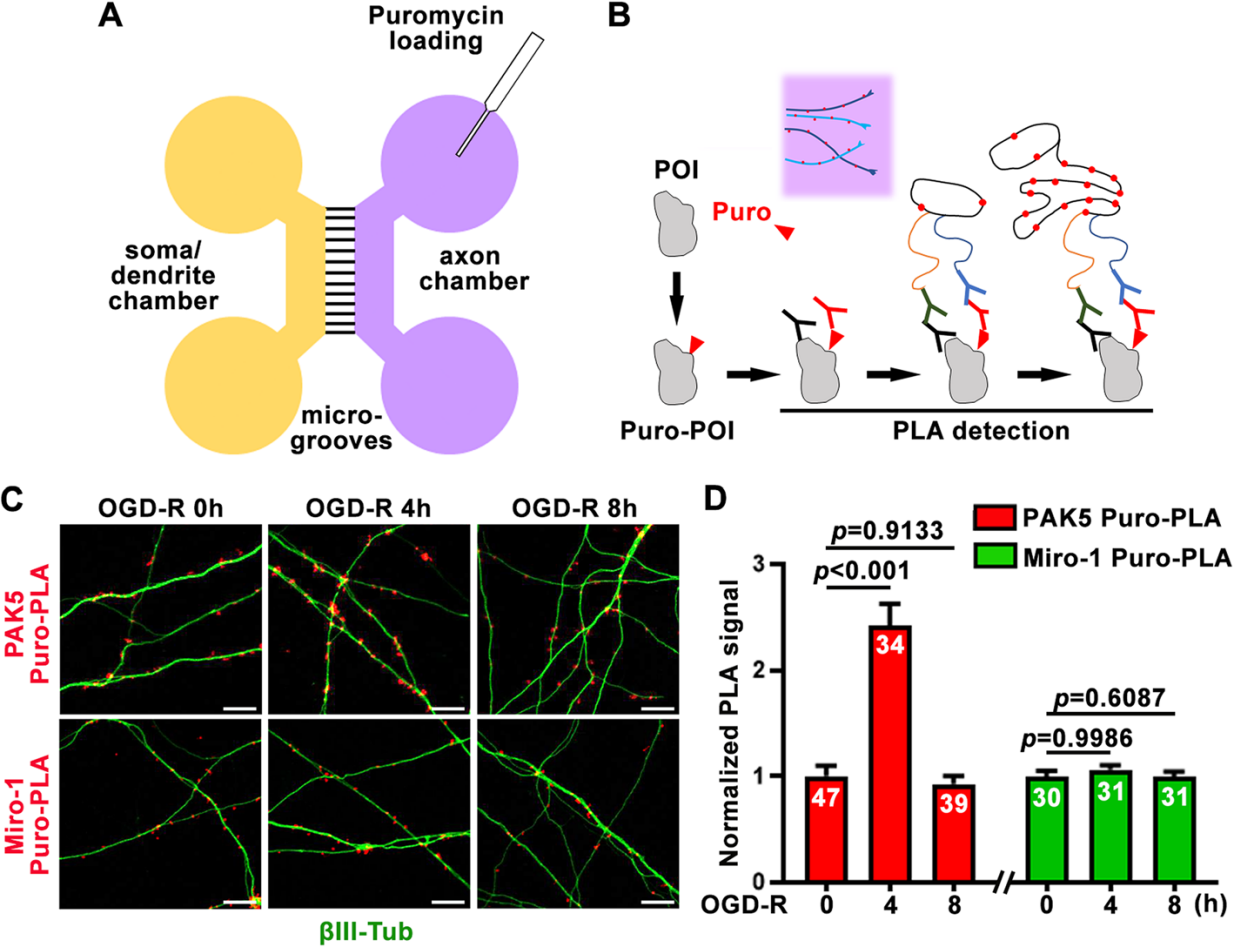
*In situ* visualization of axonal protein synthesis upon ischemic stress. **A**, **B** Metabolic labeling of puromycin in the axonal terminal chamber (**A**) and Puro-PLA assay (**B**) monitoring newly synthesized proteins *in situ*. After axon-restricted puromycin-labeling, a protein-of-interest (POI) is recognized by antibodies against puromycin (red Y) and the POI (black Y). PLA signals (red dots) are detected when PLA-plus and PLA-minus secondary antibodies (green Y and blue Y, respectively) are close enough to be ligated together and amplified by rolling-circle replication. **C**, **D**, Representative images (**C**) and quantifications (**D**) showing axonal protein synthesis in response to ischemic stress. Cortical neurons at DIV14 were subjected to OGD for 30 min, followed by reperfusion (R) for 0, 4, or 8 hours as indicated. Axons were incubated with 3 μM puromycin (Puro) for 15 min before fixation. The PAK5-Puro-PLA or Miro-1-Puro-PLA signals (red dots) along βIII-tubulin-labeled axons (green) were normalized to total βIII-tubulin signal area. Data were quantified from *n* = 30–47 images (**D**) from more than 3 chambers per condition in three independent experiments. Data were expressed as mean ± SEM and analyzed by one-way ANOVA with Tukey’s multiple comparisons test. Scale bars, 10 μm

Recovering ATP supply by reprogramming energy-sensitive signaling is thus critical to meeting increased energy demand to support CNS survival and repair. One such attractive signaling target is PAK5, a mitochondria-targeted P21-activated serine/threonine kinase of the PAK family that is expressed in developing neurons (Cotteret and Chernoff 2006; Cotteret et al. 2003; Wells and Jones 2010). Deep RNA sequencing analysis revealed that PAK5 mRNA is enriched in axons of retinal ganglion cells but declines during postnatal development (Shigeoka et al. 2016). Our recent super-resolution imaging study demonstrated that PAK5 localizes to axonal mitochondrial surface in developing neurons, and its expression and signaling decreases with neuron maturation and is undetectable in adult neurons (Huang et al. 2021). These studies raise an urgent question as to whether PAK5 signaling within axons is locally activated in response to ischemia. Given that both mRNAs of the mitochondria-targeted PAK5 and Miro-1 are found on the outer mitochondrial membrane surface (Fazal et al. 2019; Shigeoka et al. 2016), we investigated whether PAK5 and Miro-1 can be synthesized distally within axons *in situ* upon ischemic stress by combining microfluidic devices with the Puro-PLA system (tom Dieck et al. 2015) (Fig. 4A, B). A low concentration of puromycin (Puro) terminates the protein translation by the puromycylation of truncated proteins (David et al. 2012), thus allowing *in situ* detection of a newly synthesized protein-of-interest (POI) by the PLA assay using antibodies against puromycin and the POI (Fig. 4B). To model ischemia, mature cortical neurons at DIV14 were incubated in OGD media for 30 minutes, followed by reperfusion (R) for 0, 4, and 8 hours as indicated, and then by metabolic PLA labeling. To exclude diffusion of newly synthesized proteins from the soma-dendritic area, we selectively loaded 3 μM puromycin within the axonal terminal chamber to label the newly synthesized proteins along axons labeled by βIII-tubulin (Fig. 4C). Axonal PAK5 Puro-PLA signals were robustly increased at 4 hours post-OGD-R by ~ 2.42-fold (*P* < 0.001), then declined to pre-OGD treatment levels at 8 hours post-OGD-R (Fig. 4C, D). In contrast, newly synthesized Miro-1 in axons showed no significant change following the same OGD-R conditions, indicating that axonal PAK5 synthesis and local signaling is transiently activated in response to ischemia. We recently revealed that activated PAK5 signaling mediates a local phosphorylation switch that turns off SNPH-anchoring and remobilizes ischemia-damaged axonal mitochondria for replacement with healthy ones, thus reversing the injury-induced energy crisis (Huang et al. 2021).

## Discussion

In comparison to in vivo axon injury systems such as SCI, axotomy of CNS neurons on microfluidic chamber devices provides a simple and reproducible platform to study local axonal bioenergetics and signaling pathways in response to injury. It was reported that enhancing ER-mitochondria contact promotes axon regeneration (Lee et al. 2019); the impact of axonal ER dynamics and their interaction with mitochondria on local energy metabolism can also be monitored using this in vitro injury-ischemic model system. Synapse re-formation is required for reestablishing neuronal circuitry and for functional recovery during the regenerative process. However, it is technically challenging to visualize synapse reconnection in an in-vivo SCI model. A three-chamber compartmentalized microfluidic device was also developed to characterize synapse reassembly and function (Coquinco et al. 2014). Our imaging tools can be used to evaluate the roles of mitochondrial trafficking/positioning and “synaptoenergetics” during synaptic reconnection and functional recovery in injured axons. In addition to imaging local protein synthesis via metabolic labeling and PLA, several alternative labeling techniques have also been developed to visualize the dynamics of axonal mRNAs (Broix et al. 2021). Microfluidic chambers also provide a valuable imaging platform for tracking axonal mRNA transport, local protein synthesis, posttranslational modification, and signaling in axonal terminal chambers following CNS injury-ischemia. Given the versatile and affordable features of microfluidic devices combined with high levels of reliability and sensitivity of imaging tools, these injury-ischemia model systems provide ideal platforms for high-throughput screening of small molecules or drugs in reversing injury-induced energy crisis in live neurons. These platforms also benefit gene editing or reprogramming of human iPSC-derived neurons for resetting axonal energy batteries by enhancing healthy mitochondrial maintenance and/or boosting mitochondrial energy metabolism after injury and ischemia.

Considering the intricate networks in the human brain where billions of glial cells wire together with neurons (Allen & Lyons 2018), a comprehensive model for the maintenance and recovery of axonal bioenergetics after CNS injury-ischemia must include contributions by glial cells. Glial cells play protective roles in maintaining neuron survival after ischemic stress (Hayakawa et al. 2016). Oligodendrocytes serve as myelinating cells surrounding axons of the CNS, thus ideally positioning them to play a critical role in repairing injury-induced axonal energy deficits. By co-culturing oligodendrocytes with cortical axons in microfluidic devices, our recent study revealed a transcellular signaling pathway through which oligodendrocyte-derived NAD-dependent deacetylase sirtuin 2 (SIRT2) boosts axonal energy metabolism by deacetylation of mitochondrial adenine nucleotide translocases 1 and 2 (ANT1/2) (Chamberlain et al. 2021). SIRT2 is undetectable in neurons but highly expressed in mature oligodendrocytes and released within exosomes. By incubating purified exosomes within axonal terminal chambers, we detected a robust increase in local mitochondrial energy metabolism. Because glia-derived exosomes mediate the neuron-glia communication by mRNA delivery (Fruhbeis et al. 2012), it would be interesting to examine mRNA transcriptomes and local signaling pathways within axonal terminal chambers following injury-ischemia in future studies.

## Conclusions

In this methodology article, we discuss the recent advancement in the application of microfluidic devices combined with various live imaging tools to visualize CNS axon degeneration and regeneration after injury and ischemia. These state-of-the-art technologies entail cutting-edge fluorescent tools that allow us to detect dynamic changes in cellular ATP levels within axon bundles and regrowing tips, monitor axonal mitochondrial trafficking and their bioenergetic capacity, and visualize local mitochondrial protein synthesis *in situ* in response to injury and ischemic stress in mature CNS neurons. Most assays were designed based on our published studies, thus ensuring its efficacy and reliability. Establishing in vitro injury-ischemic model systems with an imaging readout provides a robust and reproducible platform for high-throughput screening of small molecules and for CRISPR/Cas9-based gene editing that enables reversing injury-induced energy crisis, reprogramming mitochondrial transport, and boosting energy metabolism within injured axons.

## Methods

### Animals

Mice were maintained in the National Institute of Neurological Disorders and Stroke (NINDS) animal facility and housed in a 12-h light/dark cycle. All animal procedures were performed according to National Institutes of Health (NIH) guidelines and were approved by the Animal Care and Use Committee of NINDS/NIDCD.

### DNA constructs

The original GFP-Mito and GO-ATeam2 constructs were gifts from R. Youle (NINDS, NIH) and H. Imamura (Kyoto University, Japan), respectively. Lentiviral constructs of GFP-Mito and GO-ATeam2 were cloned into the entry vector pDONOR221, followed by transferring into the destination lentiviral vector pHAGE-CMV-n-HA-Flag as previously reported (Zhou et al. 2016).

### Primary neuron culture

Cortical neurons were collected from E18 embryonic mice as previously described (Lin et al. 2017). Briefly, neurons were dissociated by papain (Worthington) and resuspended in a plating medium (Neurobasal medium supplemented with 2% B-27, 0.5 mM GlutaMAX, 0.1% 2-Mercaptoethanol (Thermo Fisher Scientific), 10% FBS (HyClone), and 0.25 μg/ml insulin (Sigma-Aldrich)). After 24–36 hours of growth, half of the plating medium was replaced by the same amount of neuronal feeding medium (Neurobasal medium supplemented with 2% B-27, 0.5 mM GlutaMAX, and 5 μM glia inhibitor 5-Fluoro-2-deoxyuridine). Neurons were fed every 2 days by replacing half of the medium with a fresh neuronal feeding medium. For oxygen-, glucose-deprivation (OGD) treatment of cortical neurons at DIV14, the neuronal feeding medium was replaced with oxygen-, glucose-free Neurobasal-A medium (Thermo Fisher Scientific), and quickly placed in an incubator (Thermo Fisher Scientific) with 1% O_2_, 5% CO_2_ and 94% N_2_ for 30 min. For reperfusion, the media was replaced with a normal culture medium or imaging buffer and placed in the normal incubator for the indicated time.

### Microfluidic device preparation and axotomy

A silicon wafer with a SU-8 photolithography-based pattern was used to cast the PDMS microfluidic chamber devices. Briefly, SYLGARD 184 silicone elastomer base was mixed with the curing agent at a ratio of 10:1. The PDMS was then fully mixed and poured onto the silicon wafer and then placed in a vacuum desiccator for at least 3 hours to remove the air bubbles from the PDMS. The wafer with PDMS was placed in the oven at 80 °C for 3 hours to cure. After cooling down at room temperature for 1 hour, the cured PDMS was punched out, then washed in 50% ethanol and water, dried and cleaned for 1 min in a PDC-32G plasma cleaner, and finally bonded together with plasma-cleaned coverslips before plating neurons. For the axon regeneration assay, axons in the axon terminal chambers were axotomized by vacuum aspiration at DIV12–14. To ensure complete axotomy, axon chambers were aspirated at least five times, then refilled with fresh culture medium or imaging buffer and incubated for the indicated times.

### Immunostaining and puro-PLA

Cortical neurons were fixed with PBS with 4% formaldehyde and 4% sucrose for 20 min at room temperature, then washed with PBS several times and permeabilized with PBS containing 0.1% Triton X-100 for 20 min. Neuron samples were blocked with 4% goat serum in PBS and 1% BSA for 1 hour, and incubated with a primary antibody against βIII-tubulin (1:5000, Sigma-Aldrich) diluted in blocking buffer overnight at 4 °C. After washing with PBS three times, neuron samples were fixed with a secondary antibody (Alexa 488 conjugated, Thermo Fisher Scientific) diluted in blocking buffer at room temperature for 1 hour, rewashed three times with PBS, and finally mounted with Fluoro-Gel mounting medium (Electron Microscopy Sciences) prior to imaging.

Puro-PLA was used to detect newly synthesized proteins, as previously described (tom Dieck et al. 2015) with some modifications. Three μM puromycin was loaded in the axon chamber and incubated with axons for 15 min to begin the axonal protein puromycylation process. After fixation, neurons were permeabilized with PBS containing 0.1% Triton X-100 and incubated with antibodies against puromycin (1:1000, Millipore) and PAK5 (1:200, Novus Biologicals) or Miro-1 (1:400, Sigma-Aldrich) in Duolink antibody diluent overnight at 4 °C. After washing three times with PBS and incubating with Duolink secondary antibodies (plus and minus probes) for 1 hour at 37 °C, neuron samples were incubated with Duolink PLA ligation solution for 30 min at 37 °C, washed with PBS three times, and incubated with Duolink PLA amplification solution for 100 min at 37 °C. To label the axons, PLA neuron samples were post-fixed and immunostained by an antibody against βIII-tubulin (1:5000, Sigma-Aldrich). Puro-PLA signals were measured using ImageJ (NIH).

### Live-cell imaging analysis

Transduced cortical neurons were transferred to chambers containing pre-warmed imaging buffer (Hibernate E low fluorescence medium (BrainBits) with 2% B27 and 0.5 mM GlutaMAX). A Zeiss LSM 880 Airyscan confocal microscope with a 40 × 1.3 NA oil immersion objective was used for live neuron imaging. To assess cellular ATP levels, images were collected at emissions 505–550 nm and above 545 nm within cell bodies or axons expressing GO-ATeam2 to measure ratiometric values which were generated in ImageJ (Huang et al. 2021). To analyze axonal mitochondrial transport, live images were captured along the microgrooves with 5-sec intervals, and kymographs were generated using ImageJ (Kang et al. 2008).

### Quantifications and statistical analysis

Comparisons between three or more groups were performed by one-way analysis of variance (ANOVA) with Tukey’s multiple comparisons test. Data were expressed as mean ± SEM. Differences were considered significant with *p* < 0.05. Statistical parameters, including the definitions and value of *n* (such as a total number of axons, neurons, or images), deviations, *p* values, and the types of the statistical tests, are shown in Figures and Figure Legends. Statistical analyses were performed using Prism 8 (GraphPad Software).

## Data Availability

All data generated or analyzed in this study are included in the article. Request for materials should be addressed to the corresponding author.

## Abbreviations

ATP: Adenosine triphosphate
CNS: Central nervous system
CST: Corticospinal tract
DIV: Day in vitro
FRET: Förster resonance energy transfer
OGD: Oxygen-glucose-deprivation
OGD-R: Oxygen-glucose-deprivation and reperfusion
PAK5: P21-activated kinase 5
PLA: Proximity ligation assay
PNS: Peripheral nervous system
SCI: Spinal cord injury
SNPH: Syntaphilin

## References

[CR1] Allen NJ, Lyons DA (2018). Glia as architects of central nervous system formation and function. Science..

[CR2] Bradke F, Fawcett JW, Spira ME (2012). Assembly of a new growth cone after axotomy: the precursor to axon regeneration. Nat Rev Neurosci.

[CR3] Broix L, Turchetto S, Nguyen L (2021). Coordination between transport and local translation in neurons. Trends Cell Biol.

[CR4] Chamberlain KA, Huang N, Xie Y, LiCausi F, Li S, Li Y (2021). Oligodendrocytes enhance axonal energy metabolism by deacetylation of mitochondrial proteins through transcellular delivery of SIRT2. Neuron..

[CR5] Cheng XT, Huang N, Sheng ZH (2022). Programming axonal mitochondrial maintenance and bioenergetics in neurodegeneration and regeneration. Neuron..

[CR6] Cioni JM, Lin JQ, Holtermann AV, Koppers M, Jakobs MAH, Azizi A (2019). Late endosomes act as mRNA translation platforms and sustain mitochondria in axons. Cell..

[CR7] Coquinco A, Kojic L, Wen W, Wang YT, Jeon NL, Milnerwood AJ (2014). A microfluidic based in vitro model of synaptic competition. Mol Cell Neurosci.

[CR8] Cotteret S, Chernoff J (2006). Nucleocytoplasmic shuttling of Pak5 regulates its antiapoptotic properties. Mol Cell Biol.

[CR9] Cotteret S, Jaffer ZM, Beeser A, Chernoff J (2003). p21-activated kinase 5 (Pak5) localizes to mitochondria and inhibits apoptosis by phosphorylating BAD. Mol Cell Biol.

[CR10] Cui Y, Jin X, Choi DJ, Choi JY, Kim HS, Hwang DH (2020). Axonal degeneration in an in vitro model of ischemic white matter injury. Neurobiol Dis.

[CR11] David A, Dolan BP, Hickman HD, Knowlton JJ, Clavarino G, Pierre P (2012). Nuclear translation visualized by ribosome-bound nascent chain puromycylation. J Cell Biol.

[CR12] Dieck S t, Kochen L, Hanus C, Heumuller M, Bartnik I, Nassim-Assir B (2015). Direct visualization of newly synthesized target proteins *in situ*. Nat Methods.

[CR13] Farfel-Becker T, Roney JC, Cheng XT, Li S, Cuddy SR, Sheng ZH (2019). Neuronal Soma-derived Degradative lysosomes are continuously delivered to distal axons to maintain local degradation capacity. Cell Rep.

[CR14] Fazal FM, Han S, Parker KR, Kaewsapsak P, Xu J, Boettiger AN (2019). Atlas of subcellular RNA localization revealed by APEX-Seq. Cell..

[CR15] Fruhbeis C, Frohlich D, Kramer-Albers EM (2012). Emerging roles of exosomes in neuron-glia communication. Front Physiol.

[CR16] Han Q, Xie Y, Ordaz JD, Huh AJ, Huang N, Wu W (2020). Restoring cellular energetics promotes axonal regeneration and functional recovery after spinal cord injury. Cell Metab.

[CR17] Harbauer AB, Hees JT, Wanderoy S, Segura I, Gibbs W, Cheng Y (2022). Neuronal mitochondria transport Pink1 mRNA via synaptojanin 2 to support local mitophagy. Neuron..

[CR18] Hayakawa K, Esposito E, Wang X, Terasaki Y, Liu Y, Xing C (2016). Transfer of mitochondria from astrocytes to neurons after stroke. Nature..

[CR19] He Z, Jin Y (2016). Intrinsic control of axon regeneration. Neuron..

[CR20] Huang N, Li S, Xie Y, Han Q, Xu X-M, Sheng Z-H (2021). Reprogramming an energetic AKT-PAK5 axis boosts axon energy supply and facilitates neuron survival and regeneration after injury and ischemia. Curr Biol.

[CR21] Kang JS, Tian JH, Pan PY, Zald P, Li C, Deng C (2008). Docking of axonal mitochondria by syntaphilin controls their mobility and affects short-term facilitation. Cell..

[CR22] Lee S, Wang W, Hwang J, Namgung U, Min KT (2019). Increased ER-mitochondria tethering promotes axon regeneration. Proc Natl Acad Sci U S A.

[CR23] Li S, Sheng ZH (2022). Energy matters: presynaptic metabolism and the maintenance of synaptic transmission. Nat Rev Neurosci.

[CR24] Lin MY, Cheng XT, Tammineni P, Xie Y, Zhou B, Cai Q (2017). Releasing Syntaphilin removes stressed mitochondria from axons independent of Mitophagy under pathophysiological conditions. Neuron..

[CR25] Liu H, Povysheva N, Rose ME, Mi Z, Banton JS, Li W (2019). Role of UCHL1 in axonal injury and functional recovery after cerebral ischemia. Proc Natl Acad Sci U S A.

[CR26] Lu P, Woodruff G, Wang Y, Graham L, Hunt M, Wu D (2014). Long-distance axonal growth from human induced pluripotent stem cells after spinal cord injury. Neuron..

[CR27] Nakano M, Imamura H, Nagai T, Noji H (2011). Ca(2)(+) regulation of mitochondrial ATP synthesis visualized at the single cell level. ACS Chem Biol.

[CR28] Neto E, Leitao L, Sousa DM, Alves CJ, Alencastre IS, Aguiar P (2016). Compartmentalized microfluidic platforms: the unrivaled breakthrough of in vitro tools for neurobiological research. J Neurosci.

[CR29] Sheng ZH (2017). The interplay of axonal energy homeostasis and mitochondrial trafficking and anchoring. Trends Cell Biol.

[CR30] Shigeoka T, Jung H, Jung J, Turner-Bridger B, Ohk J, Lin JQ (2016). Dynamic axonal translation in developing and mature visual circuits. Cell..

[CR31] Sun T, Qiao H, Pan PY, Chen Y, Sheng ZH (2013). Motile axonal mitochondria contribute to the variability of presynaptic strength. Cell Rep.

[CR32] Taylor AM, Blurton-Jones M, Rhee SW, Cribbs DH, Cotman CW, Jeon NL (2005). A microfluidic culture platform for CNS axonal injury, regeneration and transport. Nat Methods.

[CR33] Terenzio M, Koley S, Samra N, Rishal I, Zhao Q, Sahoo PK (2018). Locally translated mTOR controls axonal local translation in nerve injury. Science..

[CR34] Vosler PS, Graham SH, Wechsler LR, Chen J (2009). Mitochondrial targets for stroke: focusing basic science research toward development of clinically translatable therapeutics. Stroke..

[CR35] Wells CM, Jones GE (2010). The emerging importance of group II PAKs. Biochem J.

[CR36] Zheng Y, Zhang X, Wu X, Jiang L, Ahsan A, Ma S (2019). Somatic autophagy of axonal mitochondria in ischemic neurons. J Cell Biol.

[CR37] Zhou B, Cai Q, Xie Y, Sheng ZH (2012). Snapin recruits dynein to BDNF-TrkB signaling endosomes for retrograde axonal transport and is essential for dendrite growth of cortical neurons. Cell Rep.

[CR38] Zhou B, Yu P, Lin MY, Sun T, Chen Y, Sheng ZH (2016). Facilitation of axon regeneration by enhancing mitochondrial transport and rescuing energy deficits. J Cell Biol.

